# Successful Interstitial HDR Brachytherapy for Nasal Skin SCC in Xeroderma Pigmentosum: An Eight‐Year Follow‐Up Case Report

**DOI:** 10.1002/ccr3.72516

**Published:** 2026-04-08

**Authors:** Pouya Saraei, Vita Derakhshandeh, Ali Bagheri

**Affiliations:** ^1^ Department of Medical Physics, Faculty of Medicine Ahvaz Jundishapur University of Medical Sciences Ahvaz Iran; ^2^ Department of Otorhinolaryngology Iran University of Medical Sciences Tehran Iran; ^3^ Interventional Radiotherapy Ward, Department of Radiation Oncology Golestan Hospital, Ahvaz Jundishapur University of Medical Sciences Ahvaz Iran

**Keywords:** brachytherapy, radiation therapy, radiobiology, xeroderma pigmentosum

## Abstract

High‐dose‐rate interstitial brachytherapy may provide durable local control with acceptable cosmetic outcomes in carefully selected patients with xeroderma pigmentosum and cutaneous squamous cell carcinoma. This long‐term follow‐up case questions the absolute contraindication of radiotherapy in XP and underscores the importance of individualized treatment planning based on underlying DNA repair mechanisms.

## 1 Introduction

Brachytherapy (BT) delivers high doses of radiation directly to the tumor while sparing adjacent healthy tissues due to its steep dose gradient, thereby minimizing acute and late toxicities. This treatment modality may be especially beneficial for patients with radiation hypersensitivity syndromes, as it limits radiation exposure to their already vulnerable tissues, potentially reducing the risk of second primary cancers [1, 2].

Xeroderma pigmentosum (XP) is a rare autosomal recessive disorder clinically characterized by abnormal skin pigmentation and a more than 1000‐fold increased risk of non‐melanoma skin cancers (basal cell and squamous cell carcinoma) on sun‐exposed areas, resulting from a defective DNA repair mechanism, particularly in response to ultraviolet (UV) radiation [1]. XP encompasses eight complementation groups (XP‐A to XP‐G) and a variant form (XP‐V), all affecting components of the nucleotide excision repair (NER) pathway [3, 4]. While UV radiation induces single‐strand breaks and cyclobutane dimers typically repaired by NER, ionizing radiation primarily causes double‐strand breaks, repaired by non‐homologous end joining (NHEJ) and homologous recombination (HR). Therefore, XP patients are hypersensitive to UV but generally retain DNA repair capacity against X‐rays and γ‐rays. Historically, radiation therapy (RT) has been approached with caution in patients with XP due to concerns regarding toxicity and secondary malignancies [5]. However, emerging clinical experiences suggest that carefully planned radiotherapy may be feasible in selected cases in these patients [6]. Reports describing the use of BT in patients with XP are exceedingly rare, with only isolated case reports available in the literature.

This case report aims to present a unique clinical experience with the application of BT in an XP patient, contributing to the limited but growing body of literature on the safe and effective use of RT in this challenging patient population. The following sections describe our case and discuss the contrasting genomic effects of UV versus higher‐energy ionizing radiation. This case contributes to the ongoing debate on the safety of radiotherapy in XP and provides an unprecedented eight‐year follow‐up of interstitial high dose rate (HDR) BT in such a patient.

## 2 Case History

A 22‐year‐old man with clinically diagnosed XP presented in July 2017 to the Radiation Oncology Department of Ahvaz Golestan Hospital (Ahvaz Jundishapur University of Medical Sciences) with a painless nasal nodule of 2 months duration. The lesion was surgically excised, and histopathology revealed moderately differentiated squamous cell carcinoma (SCC) with positive surgical margins. His family history was notable for XP in his grandfather.

## 3 Methods

Following surgery, the lesion exhibited rapid regrowth. Due to the tumor's aggressive nature, the treating physician team initiated chemotherapy with a cisplatin and 5‐fluorouracil (5‐FU) regimen; however, disease progression persisted despite completion of the first cycle. To preserve nasal structure and function, the patient was referred to the Interventional Radiotherapy ward of Ahvaz Golestan Hospital for further evaluation.

The multidisciplinary team, including a radiation oncologist (AB) and a head and neck surgeon (VD), recommended gross tumor resection followed by immediate interstitial HDR BT, rather than a more aggressive second surgical resection aimed at achieving negative margins. This approach aimed to preserve cosmesis and nasal function while maintaining local tumor control. Tumor debulking was performed to achieve cytoreduction, lower the clonogen burden, potentially reduce the radiation dose required for tumor eradication, and minimize the risk of catheter displacement during BT due to anticipated tumor necrosis. Following tumor debulking, HDR interstitial BT was delivered with definitive intent rather than as an adjuvant boost, aiming to achieve durable local control while preserving nasal anatomy and cosmetic outcome.

**FIGURE 1:**
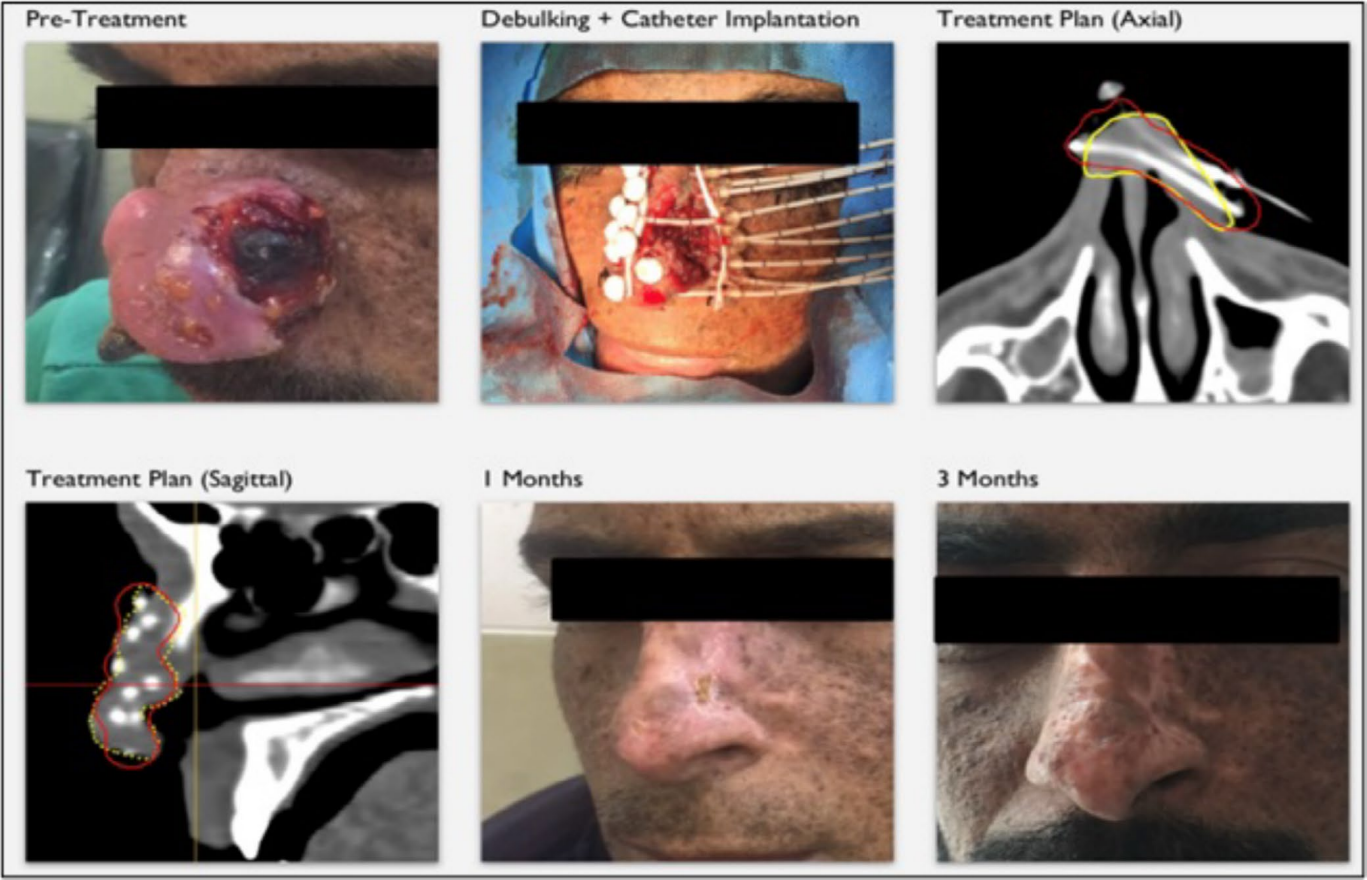
Pre‐BT, post‐debulking with catheter implantation, treatment planning, and short‐term follow‐up. Yellow contour: Planning Target Volume (PTV), Red contour: 100% isodose line.

BT was preferred over external beam radiation therapy (EBRT) because of concerns regarding radiation hypersensitivity in this patient and to potentially reduce the risk of second primary cancers. The patient provided written informed consent for publication of anonymized clinical data and accompanying images. According to institutional policy, ethics committee approval is not required for single‐patient case reports. Technical details of the interstitial BT procedure are presented in Table 1, while Figure 1 illustrates images obtained before BT, after tumor debulking with catheter implantation, treatment planning, and during short‐term follow‐up. The achieved CTV V100% of 89% reflects anatomical constraints of the nasal region and intentional dose shaping to balance adequate target coverage with preservation of surrounding normal tissues and cosmetic outcome.

**TABLE 1:** Technical details of interstitial BT delivered in this patient.

Brachytherapy	HDR
Source	Cobalt‐60
Total Prescribed Dose	36 Gy
Number of Fractions	12
Treatment Schedule	Twice a day (bid)
CTV Volume	9.74 cc
Number of Implanted Catheters	9
CTV D_90%_	3 Gy (Each fraction)
CTV V_100%_	89%
DNR	26%

Abbreviations: CTV, Clinical target volume; D_90%_, The minimum dose received by the most exposed 90% of the CTV; DNR, Dose non‐uniformity ratio (= V_150%_/V_100%_); HDR, High dose rate; V_100%_, The volume of the CTV receiving 100% of the prescribed dose; V_150%_, The volume of the CTV receiving 150% of the prescribed dose.

## 4 Conclusion and Results

**FIGURE 2:**
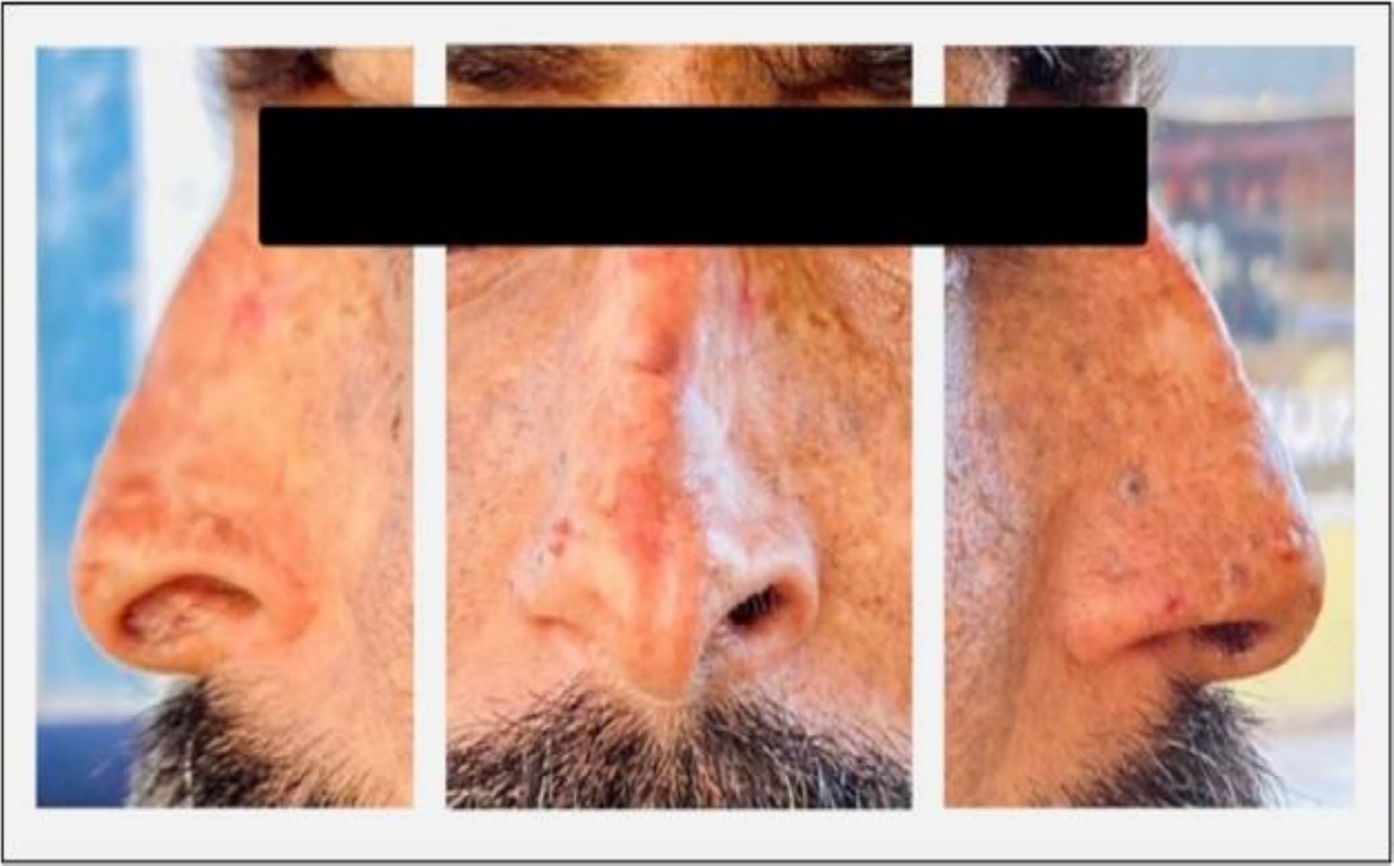
Eight‐year follow‐up showing tumor‐free status with preserved nasal structure and acceptable cosmesis.

During the first 2 years following BT, the patient reported localized sharp pain at the nasal tip upon palpation, but experienced no episodes of epistaxis, nasal dryness, rhinorrhea, or respiratory distress. At the most recent follow‐up, 8 years post‐treatment (July 2025), the patient remains tumor‐free, with preserved nasal structure and satisfactory cosmetic outcome (Figure 2). During long‐term follow‐up, no late tissue necrosis, chronic ulceration, or radiation‐induced secondary malignancies within the treated field were observed.

This case supports the potential role of HDR interstitial BT as a safe and effective treatment for selected XP patients with localized skin SCC. While our findings suggest that XP‐related UV hypersensitivity may not necessarily translate to ionizing radiation intolerance, further multicenter data are needed before altering established contraindications. This report emphasizes the need for individualized treatment planning and cautious optimism when applying RT in genetic hypersensitivity syndromes.

## 5 Discussion

This case illustrates the challenges of treating cutaneous malignancies in XP patients, where concerns about radiation hypersensitivity have traditionally limited the use of RT. Our findings suggest that BT can achieve durable local control with minimal toxicity and long‐term acceptable cosmetic outcomes.

Although patients with XP are highly sensitive to UV radiation and also were thought to be at increased risk of DNA damage from ionizing radiation, many case reports and case series have demonstrated that RT can be safely administered in this patient population [7, 8, 9].

BT and modern EBRT technologies when carefully planned, can deliver localized conformal tumoricidal doses to the target volume, while sparing surrounding healthy tissues, thereby reducing treatment‐related early and late treatment toxicities even in individuals with hypersensitivity syndromes to ionizing radiation. Compared with previously reported cases of radiotherapy in patients with XP, which predominantly involve external beam techniques and shorter follow‐up durations, this case is notable for the use of interstitial HDR BT and an eight‐year disease‐free interval. This extended follow‐up provides rare long‐term clinical insight into the tolerability and durability of localized BT in this high‐risk population.

The cells deficient in NER subtypes, particularly XP‐C and XP‐D, exhibit pronounced sensitivity to UVA and UVB radiation [10]. In contrast, photon‐induced DNA lesions primarily comprise single‐strand breaks (SSBs), abasic sites, and oxidized bases, which are repaired by the base excision repair (BER) machinery [11]. Moreover, ionizing radiation induces double‐strand breaks (DSBs), which are repaired by non‐homologous end joining (NHEJ) and homologous recombination repair (HRR), each mediated by distinct protein complexes [12]. Because the mutational defects in XP affect NER rather than BER, NHEJ, or HRR, these patients while hypersensitive to UV radiation retain largely functional repair machinery for ionizing radiation–induced DNA damage. However, as a single‐patient observation, this case cannot establish generalizable safety thresholds. Larger case registries and prospective collaborations are needed to refine RT protocols for XP.

## Author Contributions

**Pouya Saraei:** writing – original draft, writing – review and editing. **Vita Derakhshandeh:** writing – original draft, writing – review and editing. **Ali Bagheri:** supervision, writing – original draft, writing – review and editing.

## Funding

The authors have nothing to report.

## Ethics Statement

As a single‐case report with the patient's signed consent, no other ethical review was required.

## Consent

We ensure that a statement of consent to publish these findings and images was gathered from the patient.

## Conflicts of Interest

The authors declare no conflicts of interest.

## Data Availability

The data that support the findings of this study are available from the corresponding author upon reasonable request.
